# Natives Engages in Alzheimer’s Research (NEAR)

**DOI:** 10.1002/alz.091030

**Published:** 2025-01-09

**Authors:** Marija Bogic

**Affiliations:** ^1^ Washington State University, Seattle, WA USA

## Abstract

**Background:**

The Native Hawaiian/Pacific Islander (NHPI) and American Indian/Alaska Native (AI/AN) populations are rapidly aging but there is limited data on Alzheimer’s disease and related dementias (ADRD). Despite the high prevalence of ADRD risk factors, the healthcare systems serving these communities are inadequately equipped to address the economic and social impact of ADRD. NEAR aims to reduce ADRD‐related health disparities by collaborating with a wide network of community and academic partners to gain a better understanding of the disease, implement interventions, and mitigate its impact on these communities.

**Method:**

NEAR is the only P01 focusing on AI/ANs or NHPIs and ADRD‐related health disparities since the NIA began documenting P01s in 1984. Based at Washington State University, NEAR includes collaborators from the University of Miami, Brigham Young University, University of Hawai`i, University of Washington, University of Minnesota, and University of Colorado, along with 35 community partners. The program consists of 3 culturally‐informed projects, supported by an Administrative Core, Recruitment and Engagement Core, Biospecimen Core, and Research Methods Core. Notably, NEAR involves 12 AI/AN or NHPI scientists and all Projects and Cores are led by a Native researcher. Indians Transforming Alzheimer’s Care Training is a group‐randomized trial to improve ADRD diagnosis and care for AI/AN elders through culturally‐informed provider training. ‘IKE Kupuna is a randomized controlled trial testing the effectiveness of Hula in preventing cognitive decline in NHPI elders. Cognition After Obstructive Sleep Apnea Treatment Among Native American People is an observational study that screens for obstructive sleep apnea and cognitive impairment on 2 reservations, followed by a trial of motivational interviewing to improve adherence to sleep apnea treatment and improve or preserve cognitive function.

**Result:**

There are no results since this is a grant abstract.

**Conclusion:**

The decentralized model and collaboration with a diverse group of AI/AN and NHPI scientists, along with world‐class ADRD experts, has enabled NEAR to create unique scientific resources and a large network of academic and community partnerships. This integrated effort holds great promise for improving the diagnosis and quality of ADRD care while advancing the scientific field.

